# Meta-analysis of RNA-seq expression data across species, tissues and studies

**DOI:** 10.1186/s13059-015-0853-4

**Published:** 2015-12-22

**Authors:** Peter H. Sudmant, Maria S. Alexis, Christopher B. Burge

**Affiliations:** Department of Biology and Biological Engineering, Massachusetts Institute of Technology, Cambridge, MA 02142 USA; Program in Computational and Systems Biology, Massachusetts Institute of Technology, Cambridge, MA 02142 USA

## Abstract

**Background:**

Differences in gene expression drive phenotypic differences between species, yet major organs and tissues generally have conserved gene expression programs. Several comparative transcriptomic studies have observed greater similarity in gene expression between homologous tissues from different vertebrate species than between diverse tissues of the same species. However, a recent study by Lin and colleagues reached the opposite conclusion. These studies differed in the species and tissues analyzed, and in technical details of library preparation, sequencing, read mapping, normalization, gene sets, and clustering methods.

**Results:**

To better understand gene expression evolution we reanalyzed data from four studies, including that of Lin, encompassing 6–13 tissues each from 11 vertebrate species using standardized mapping, normalization, and clustering methods. An analysis of independent data showed that the set of tissues chosen by Lin et al. were more similar to each other than those analyzed by previous studies. Comparing expression in five common tissues from the four studies, we observed that samples clustered exclusively by tissue rather than by species or study, supporting conservation of organ physiology in mammals. Furthermore, inter-study distances between homologous tissues were generally less than intra-study distances among different tissues, enabling informative meta-analyses. Notably, when comparing expression divergence of tissues over time to expression variation across 51 human GTEx tissues, we could accurately predict the clustering of expression for arbitrary pairs of tissues and species.

**Conclusions:**

These results provide a framework for the design of future evolutionary studies of gene expression and demonstrate the utility of comparing RNA-seq data across studies.

**Electronic supplementary material:**

The online version of this article (doi:10.1186/s13059-015-0853-4) contains supplementary material, which is available to authorized users.

## Background

Phenotypic differences among species are often driven by evolutionary adaptations in gene expression, yet many developmental programs and pathways are deeply conserved. Gene expression among homologous genes across vertebrate species and tissues has been explored using microarray [1] and RNA-sequencing (RNA-seq) [2–4]. All of these studies concluded that gene expression was more similar between homologous organs of different species than between different organs of the same species. This result has been interpreted as a reflection of evolutionarily conserved transcriptional programs driving the production of major proteins that define specific organs, such as heart, lung, or liver. This result supports the accepted idea that non-human vertebrate models, such as rodents, serve as useful models of the physiology of particular human organs, despite tens of millions of years of evolutionary divergence. Recently, however, a study assessing 13 human and mouse tissues challenged this result, concluding that different organs within a species are more similar in gene expression than homologous organs in different species [5, 6].

Reconciling the disparate conclusions of Lin et al. [6] with previous studies is made challenging by the many technical aspects of assessing and comparing expression profiles between samples of different organs and species collected by different studies. Indeed, the study design of this paper has been criticized, and batch effects were proposed as a potential source of the observed clustering patterns [7]. While RNA-seq has been heralded as being free from many of the technical biases associated with microarray-based expression analyses, numerous technical variables have been recognized that can impact downstream analyses. For instance, different library construction protocols can yield different sequence coverage, complexity, evenness, and expression level estimates [8]. Differences exist among sequencing platforms and even between different versions of the same platform, for example, Version 3 of the Illumina Hi-Seq platform yields better representation of higher C+G sequences than Version 2 [9]. Specific choices of experimental subjects and sample isolation and handling are also relevant. Gene expression patterns vary with the age of an individual [10], and the post-mortem interval prior to RNA extraction significantly impacts the integrity of the isolated sample [11]. Dissections may also include varying amounts of surrounding tissue, and different preservation methods can impact RNA quality [12]. Finally, interspecies studies of gene expression have interrogated different organs and tissues from species of varying evolutionary divergences, spanning from one to hundreds of millions of years. Patterns of interspecies gene expression conservation are likely to differ among organs and may exhibit varying dependence on evolutionary distance.

To attempt to better understand gene expression evolution and what conclusions are universal among the various studies, we performed a meta-analysis of four datasets encompassing 6–13 tissues from 11 vertebrate species [2, 3, 6], supplemented by 51 human tissues sequenced by the GTEx consortium [13]. We found that clustering by species or tissue was predictable dependent both on the subset of tissues selected and the divergence time of the species analyzed.

## Results

### Interspecies clustering by tissue is the predominantly observed pattern among various studies under various distance metrics and normalization methods

**Fig. 1:**
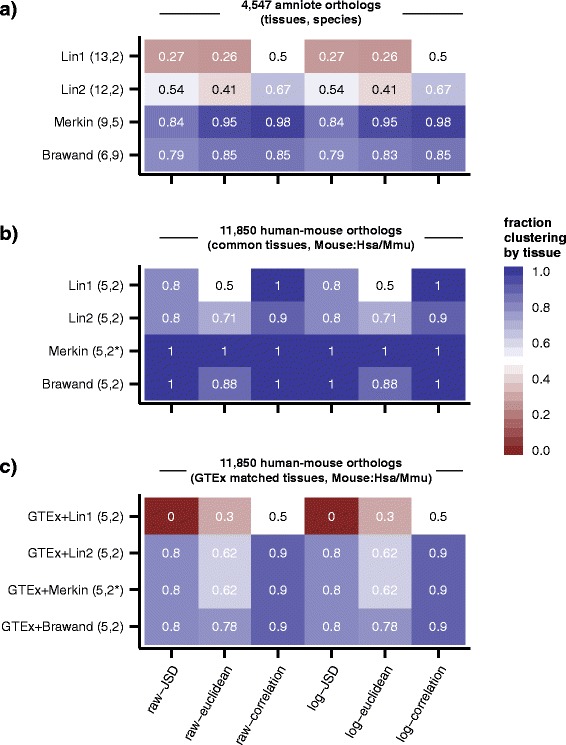
Samples cluster largely by tissue across various studies and using a variety of distance metrics. Heat maps of the extent of interspecies tissue clustering, defined as the proportion of samples clustering most closely with a sample of a homologous tissue in a different species. Each heat map row represents a single set of samples assessed using various normalization methods and distance metrics (columns). **a** Representative samples (one per tissue/species) from four datasets spanning 11 species and from 6–13 tissues assessed over 4,547 common amniote orthologs. Lin1 and Lin2 represent identical library preparations sequenced at different times. **b** Mouse and human (*macaque was substituted for Merkin as no humans were assessed) samples assessed among the five tissues common to all studies (brain/cerebellum, testis, heart, kidney, and liver) over 11,850 human-mouse orthologs. **c** Mouse samples from each study paired with matched human tissues sequenced as part of the GTEx study assessed over 11,850 human/mouse orthologs in five common tissues

To assess patterns of clustering among tissues and species, we reanalyzed RNA-seq data from Merkin et al. [3] (nine tissues, five species), Brawand et al. [2] (six tissues, nine species), and Lin et al. [6] (referred to as Lin1, 13 tissues, two species) in addition to resequencing data from 12 of the original Lin et al. library preparations (referred to as Lin2, 12 tissues, two species) (Table 1). Each of these datasets was mapped using a common pipeline and read counts were assessed over a common set of either amniote or human-mouse orthologs. While several methods for RNA normalization have been proposed [14], we selected the trimmed mean of M-values (TMM) normalization method [15], which normalizes the expression values of a set of experiments to log fold changes against an arbitrary chosen reference sample excluding outliers. We chose this method for several reasons, including its relatively common use and simplicity, and its numerous advantages over similar methods [14]. We then computed the pairwise distance among samples using raw TMM normalized gene counts or log-normalized TMM counts as measured by three different distance metrics. While several distance metrics have been proposed exhibiting various strengths, we retained three of the most commonly used, Pearson correlation, Euclidean distance, and Jensen–Shannon Divergence (JSD). We counted the number of samples that clustered most closely with a homologous tissue sample from a different species (T), and the number that clustered with a different tissue from the same species (S), and determined the fraction clustering by tissue as T/(T+S) (Fig. 1a). As was previously observed in the Brawand et al. and Merkin et al., studies, most samples in these datasets clustered by tissue (79–98 %), irrespective of the normalization or distance metric applied. Curiously, the Lin1 and Lin2 datasets, sharing 12 common samples and identical in all aspects but sequenced at different times, exhibited vastly different sample clustering. Among the various normalization and distance metrics, 26–50 % of samples clustered by tissue in the original Lin et al. dataset compared to 41–67 % of samples in the resequenced dataset.

**Table 1:** Summary of datasets and tissue samples analyzed in this study

Dataset(s)	Tissues	Species
Merkin	brain	chicken
	colon	cow
	heart	mouse
	kidney	rat
	liver	macaque
	lung	
	skeletal muscle	
	spleen	
	testes	
Brawand	brain	chicken
	cerebellum	platypus
	heart	opossum
	kidney	mouse
	liver	macaque
	testes	orangutan
		gorilla
		chimp
		human
Lin1/Lin2	adipose	human
	adrenal	mouse
	brain	
	heart	
	kidney	
	liver	
	lung	
	ovary^a^	
	pancreas	
	sigmoid colon	
	small bowel	
	spleen	
	testes	
GTEx	51 profiled tissues^b^	human

^a^ not included in Lin2, ^b^ full list of tissues in Fig. 4

To resolve whether the particular choices of tissues or species were responsible for the observed clustering trends, we focused on the five tissues common to all four datasets (brain, heart, liver, kidney, and testes) and on the human and mouse species (or macaque and mouse in the case of Merkin et al., which did not analyze human) in 11,850 human/mouse orthologs. Previous studies have noted that these five tissues exhibit quite distinct expression and proteomic profiles [16–18]. Strikingly, when this subset of tissues and species was assessed, clustering by tissue exceeded 50 % for all datasets, irrespective of normalization or distance metric, with Lin2, Merkin, and Brawand all exhibiting >71 % of samples clustering interspecies by tissue (Fig. 1b). Substituting matched human tissues assessed by GTEx for the human/macaque tissues assessed by each of these studies resulted in a similar proportion of samples clustering by tissue for Lin2, Merkin, and Brawand, but dramatically reduced the fraction of samples clustering by tissue in Lin1 (Fig. 1c). This observation suggests that some aspect of the sequencing performed in the original Lin study differed from sequencing performed later by these authors or by the other studies.

### Tissues assessed by Lin et al. are more similar than those assessed by previous studies

**Fig. 2:**
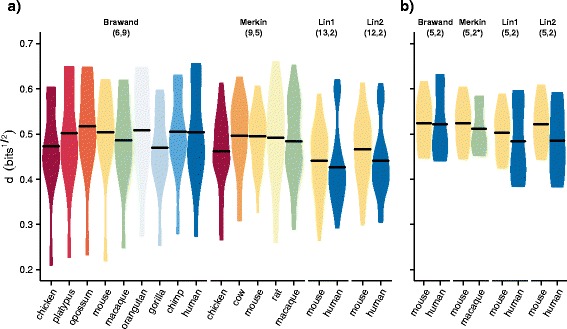
Typical inter-tissue distances are similar among most datasets, with Lin et al. tissues exhibiting smaller distances. The distribution of all pairwise distances among tissues within an individual species for various datasets is shown with the square root of the Jensen–Shannon Divergence (JSD^½^) as the distance metric throughout. **a** The distances among tissues calculated for all interspecies datasets using 4,547 orthologs common to the 11 amniotes assessed. *Black bars* indicate the mean. **b** The distribution of distances among the five tissues common to all studies assessed (brain/cerebellum, testis, heart, kidney, and liver), for mouse and human/macaque species over 11,850 human/mouse orthologs

While analyses of the both the original and resequenced Lin et al. data exhibited noticeably less interspecies clustering by tissue, considering the subset of the Lin data from the five tissues common to all studies recapitulated the previously observed pattern of interspecies clustering of tissues. We plotted the distribution of intraspecies JSD distances among tissues for all studies (Fig. 2a), and found the Lin tissues to be more similar on average with a mean of 0.43 and 0.45 bits^½^ for Lin1 and Lin2 respectively compared to 0.49 bits^½^ each for Brawand and Merkin. However, considering only the subset of five common tissues this difference was substantially diminished, though still lower in both the Lin1 and Lin2 datasets (Lin1 and Lin2 0.49 and 0.50 bits^½^ respectively compared to 0.52 bits^½^ each for Merkin and Brawand) (Fig. 2b). Here and for further analyses we used log-JSD distance because of its information theoretic properties.

### Inter-study distances between homologous tissues are generally less than intra-study distances among different tissues

**Fig. 3:**
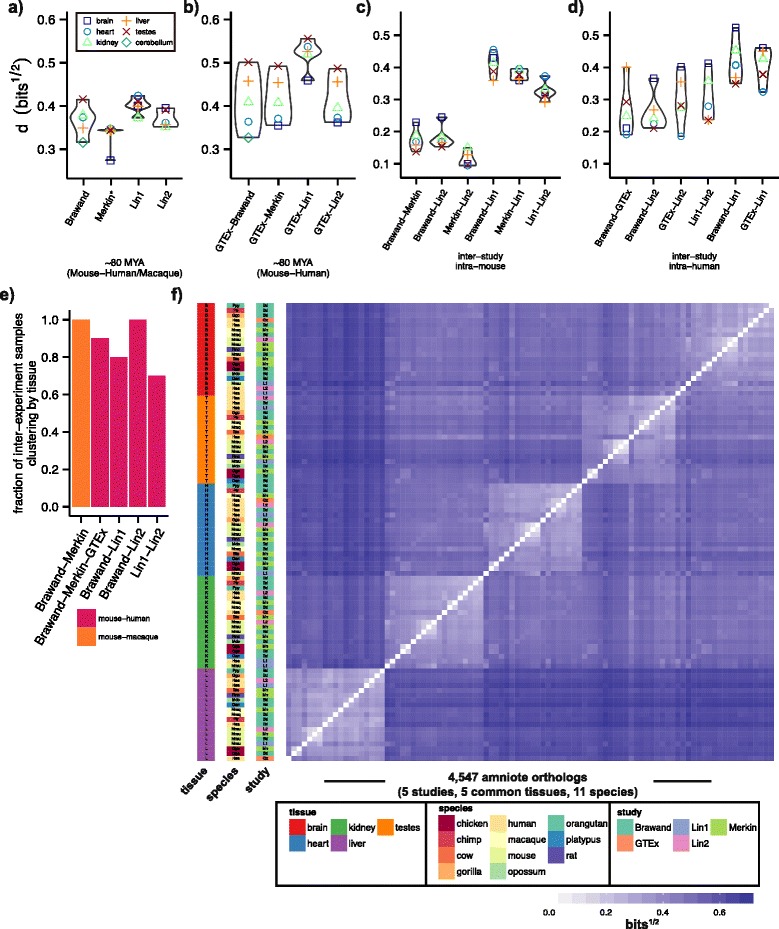
Inter-study distances between homologous tissues are small enough to enable meta-analysis, yielding clustering by tissue across species and studies. **a** The distance (JSD^½^) between matched mouse and human/macaque tissues within studies. **b** The distance (JSD^½^) between matched mouse and GTEx human samples. The inter-study, intraspecies distances among (**c**) mouse tissues and (**d**) among human tissues. **e** The fraction of samples clustering most closely with a sample of the same tissue considering only inter-study relationships. **f** Heat map hierarchical super-clustering of 94 samples encompassing five shared tissues, five datasets, and 11 different species

The common tissues sequenced in multiple human and mouse biological samples by each of the studies provided a unique opportunity to assess the impact of inter-study technical variability. We first compared the interspecies distances between matched mouse and primate tissues common to the four datasets. We observed similar distributions of distances in all studies, ranging from 0.33 to 0.40 bits^½^ (Fig. 3a). Pairing the mouse tissues of each study with matched GTEx human samples yielded an increased interspecies tissue distance (Fig. 3b). However, this increase was >25 % greater for Lin1 than for Merkin, Brawand, or Lin2 (0.52 bits^½^ compared to ~0.41 bits^½^). The relative ordering of interspecies distances between matched tissues was identical, and the magnitudes similar, among Merkin, Brawand, and Lin2, with brain tissues exhibiting the least distance between species and testes samples the most.

We next assessed the distances between identical tissue/species pairs from different studies (Fig. 3c). Notably, for pairs of mice sequenced by the Brawand, Merkin, and Lin2 studies, the mean inter-study distance between matched mouse tissues (ranging from 0.11 to 0.18 bits^½^) was less than the mean intra-study interspecies distance for the same tissues and studies (which ranged from 0.33 to 0.37, Fig. 3a). This result implies that RNA-seq data of this period can reasonably be pooled among studies for meta-analyses of gene expression, in contrast to the situation for most previous genome-wide expression profiling technologies. However, the mouse tissues from the Lin1 study were a distinct outlier in this regard, exhibiting an almost 3-fold increase in the mean inter-study distance to identical mouse tissues from other studies. The inter-study comparisons of human tissues were more variable (Fig. 3d), likely owing to the many challenges associated with analyzing RNA from human tissues. Comparisons to Lin1 human tissues yielded somewhat higher mean distance between homologous tissues.

### Common tissues exhibit interspecies clustering when exclusively comparing samples between studies

For mouse and human or macaque, we grouped studies and identified the closest clustering sample pairs, excluding any sample pair sequenced by the same study. We used this analytical approach to identify trends in the data that were robust to the technical variation that exists between studies. We additionally combined the Brawand, Merkin, and GTEx studies, considering inter-study distances between the Brawand and Merkin mouse samples and the Brawand and GTEx human studies. Notably, interspecies clustering by tissue was the dominant pattern observed among all inter-study combinations, with Brawand, Merkin, Lin2, and GTEx combinations clustering by tissue at least 90 % of the time (Fig. 3e). Inter-study comparisons including Lin1 also exhibited clustering mostly by tissue, ranging from 70 to 80 %, with the Lin1–Lin2 pairing exhibiting the least interspecies clustering. We then pooled and hierarchically clustered 95 samples from the five common tissues assessed among Brawand, Merkin, Lin1, Lin2, and GTEx, representing 11 different species (Fig. 3f). This super-clustering of various tissues, species, and independent studies yielded perfectly consistent clustering by tissue.

### Clustering by species or tissue is predictably dependent on the subset of tissues selected and the divergence times of the species analyzed

Despite the robust interspecies clustering by tissue observed among the five tissues common to all studies analyzed, we observed that the complete set of tissues assessed in both Lin1 and Lin2 were more similar to each other than the sets of tissues chosen by either Merkin or Brawand (Fig. 2). Technical aspects of the Lin et al. study, such as the tissue harvesting technique or post-mortem interval, could drive this increased similarity, or these particular tissues may simply be inherently more similar in their expression patterns. If the latter were true then one might expect that these tissues would also appear more similar to each other in independent datasets. Furthermore, if the distances between these particular tissues did not exceed the typical interspecies distance between homologous tissues for a particular divergence time, then the samples should cluster by species rather than by tissue.

**Fig. 4:**
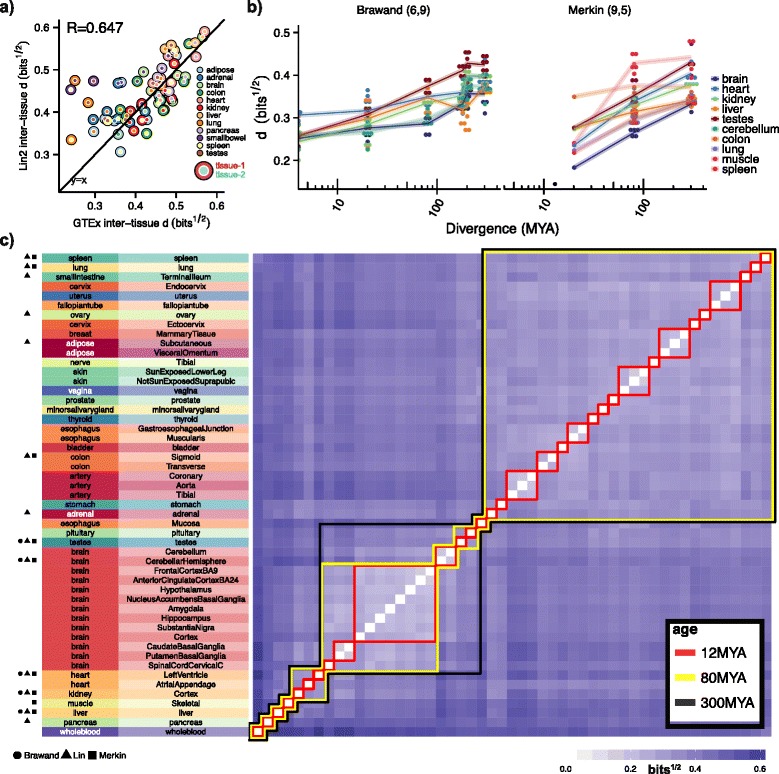
Clustering by species or tissue is predictably dependent on the subset of tissues selected and the divergence times of the species analyzed. **a** Inter-tissue distance (JSD^½^) between Lin2 and GTEx human samples overlaid with line y = x. **b** The distance (JSD^½^) between matched tissues among species plotted as a function of evolutionary time for all tissues and species assessed in the Brawand and Merkin studies (n = 43). **c** Clustering heat map of 51 human tissues sequenced by GTEx. *Distances* represent the mean inter-tissue distance calculated among three individuals. *Colored boxes* indicate the flat clusters (groupings) formed for distance cutoffs corresponding to the mean interspecies tissue distance at specific divergence times. Tissues within a cluster have an inter-tissue distance lower than the mean interspecies distance between matched tissues. *MYA* million years ago

We sought to test this hypothesis by first comparing the inter-tissue distances among the 12 human Lin2 samples (66 pairs, i.e., $$ \left(\begin{array}{c}\hfill 12\hfill \\ {}\hfill 2\hfill \end{array}\right) $$) to the inter-tissue distances observed in GTEx samples (Fig. 4a). These inter-tissue distances were highly correlated between the two studies (R = 0.65), falling on the line y = x, suggesting the tissues selected by Lin et al. were inherently more similar biologically, and not for technical reasons, though GTEx tissues tended to be slightly more similar to one another on average (mean GTEx–Lin2 inter-tissue distance of −0.023). We next attempted to model how this tissue similarity affects interspecies clustering analyses by assessing the phylogenetic relationships among all species pairs assessed by Merkin and Brawand (*n* = 43 pairs) and calculating the mean distance between matched tissues at all species-pair divergence times (Fig. 4b). As has been previously observed [2], the interspecies distance between matched tissues increases as a function of evolutionary distance. The mean distance between matched tissues from a pair of species can thus be estimated from their divergence time. We compared the inter-tissue distances among 153 human samples encompassing 51 tissues sequenced by GTEx and hierarchically clustered these based on the mean pairwise distances between tissues from different individuals (Fig. 4c). The clustering relationships that emerged among human tissues are consistent with previous observations [16] and “sub-tissues” clustered together as expected. For the ~12, ~80, and ~300 million year species split-times we then projected the mean interspecies tissue divergences onto the GTEx inter-tissue relationships, identifying clusters of tissues within which, for a given divergence time, the inter-tissue distance was lower than the mean interspecies distance between matched tissues. The distance between these clusters exceeded the mean interspecies distance between matched tissues for a particular divergence time. Notably, we observed that for Brawand and Merkin, the set of tissues chosen fell largely into independent clusters for all three divergence times, while more than half the Lin tissues fell into single clusters with inter-tissue distances less than the average 80 million or 300 million year divergence distance. Thus, the patterns of interspecies and intraspecies clustering observed in each of the Merkin, Brawand, and Lin2 datasets match perfectly to a simple model based on the typical divergence rates of tissues during mammalian evolution.

### Intraspecies inter-tissue distances are conserved and isomorphic

**Fig. 5:**
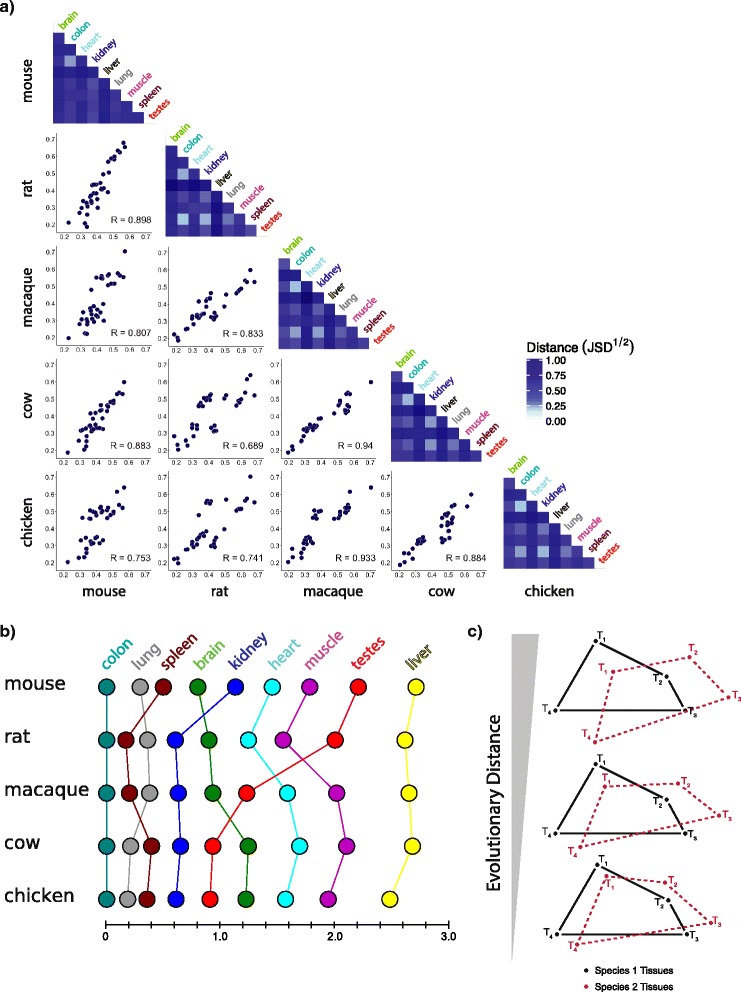
Inter-tissue distance matrices are conserved across species. **a** Within-species inter-tissue distances (JSD^½^) are plotted between pairs of species. Heat maps along the diagonal show the magnitude of inter-tissue distances for a particular species. **b** The relative ordering and magnitude of distances between tissues within species are shown. Distances between nodes along the x-axis represent the distance between the tissues at nodes i and i + 1 respectively. Vertical lines connecting homologous tissues are for visualization purposes. **c** A schematic model of the relationship between interspecies and intraspecies tissue distances as a function of evolutionary divergence. The *red* and *black graphs* represent the distances between four tissues (T_1_ to T_4_) in two different species. The graphs were drawn such that the mean distance between tissues in a species exceeds the interspecies distance between homologous tissues by roughly the observed ratio for a human–macaque comparison (*bottom*), a human–mouse comparison (*middle*), or a human–chicken comparison (*top*), and to agree with the result above that relative tissue differences tend to be conserved across species

An implicit assumption in our simple predictive model of interspecies and intraspecies tissue clustering is that the distances among tissues within a species remain relatively constant over evolutionary time. To explore this issue, we compared the correlation among intraspecies, inter-tissue distances between pairs of species (Fig. 5a). The distances between tissues within species were highly correlated with each other (mean R = 0.86), suggesting that inter-tissue distances are indeed largely isomorphic among mammalian species. We next specifically compared the relative ordering of intraspecies tissue distances by sorting tissues within species based on their distances from one another, using colon as a reference (Fig. 5b). Among species, these tissue orderings were highly similar, with one exception being the relative positioning of the testis. Testis placed as the fifth or sixth most distant tissue from colon in chicken, cow, and macaque. In mouse and rat, testis placed eight most distant from colon. Gene expression in the testis has been previously shown to be the most rapidly divergent throughout evolution [2], a result that we confirmed in our interspecies comparisons of matched tissues (Fig. 4b). The way in which distances between tissues and species diverge over time is visually represented in Fig. 5c. This diagram summarizes our findings that the divergence in species-specific expression is generally smaller than the typical inter-tissue distances and that the distance relationships between tissues are largely conserved over time.

## Discussion and conclusions

While vertebrate species differ in many phenotypic traits, they share similar body plans and many of the same organs and tissues. Evolutionary gene expression studies serve to provide insights into the molecular basis of these conserved and divergent phenotypes. Here, we performed a meta-analysis of four different studies to assess the relative differences in gene expression among different samples, tissues, species, and studies. We affirm that in every study, including the resequenced Lin et al. data, the majority of samples clustered with homologous tissues of different species rather than with different tissues of the same species, a pattern that held when using different normalization and distance metrics. This observation is consistent with the idea that many developmental gene expression programs are conserved across mammals [1–3] and supports the utility of rodents as models of human tissue physiology.

Furthermore, we found that the technical variation between RNA-seq studies was in general less than the biological variation between different tissues of the same species or between matched tissues of different species (Fig. 3a-e), implying that comparisons of samples from different RNA-seq studies can yield insights into questions about mammalian tissue biology. This finding is encouraging in light of the many potential technical differences between such studies. While batch effects must always be taken into account during study design, many RNA-seq datasets appear robust to inter-study technical variation, a property that generally does not hold for microarray-based gene expression analysis. For instance, both the relative ordering and magnitude of intraspecies and interspecies gene expression distances were largely reproducible among studies considered here. We expect this property to hold in future studies, as long as the technical variation falls below the typical expression variation among the samples analyzed. A study assessing a set of very similar tissue subtypes, for instance, may be a poor candidate for meta-analysis owing to the small variation amongst samples.

Because some groups of tissues are inherently more similar to each other, the choice of tissues can impact conclusions regarding clustering patterns. Evolutionary divergence in expression has been assessed [2], but our study is the first to compare within-species, inter-tissue distances to expected interspecies distances between matched tissues. The particular tissues selected by the Merkin and Brawand studies were more divergent from each other than those chosen by Lin and colleagues. As a result, while samples from homologous tissues of different species predominantly clustered together in Merkin and Brawand samples, just 54 % of samples from the Lin study clustered together by tissue rather than species (Fig. 1a). The underlying biological basis for diversity in gene expression between tissues is likely to be complex. Gene expression differences may result from differences in the cell type composition of individual tissues or from general or cell-specific changes in gene expression levels. Single cell transcriptomics approaches will help resolve these possibilities, while other approaches will be needed to distinguish whether differences result from changes in transcription or in mRNA stability.

Our clustering approach exclusively used pairwise distances between samples, differing from some previous studies. Other studies have relied primarily on PCA, Principal Component Analysis, which assesses variance across the dataset as a whole, and depends more on the overall makeup of the input samples. PCA and similar dimension reduction techniques are valuable approaches. We performed multidimensional scaling on data from all five datasets, yielding a pattern which supports interspecies clustering of matched tissues (Additional file 1: Figure S1). However, these approaches have some drawbacks. In PCA for instance, if most tissues assessed are highly similar, falling below the mean matched-tissue distance for a particular evolutionary divergence time, then the vector explaining the largest component of variance will generally separate the species, even if many individual tissues have higher similarity to the corresponding tissue of another species than to any tissue in the same species (Additional file 2: Figure S2). We are therefore cautious about drawing broad conclusions based on PCA of a collection of samples, particularly when considering just one or a few components.

Finally, using the relationships among 51 tissues sequenced by the GTEx consortium and the expected interspecies distance between matched tissues at specific divergence times, we found that the extent of interspecies and intraspecies tissue clustering was largely predictable (Fig. 4c). This approach implicitly assumes that the relative distances between tissues within an organism tend to be conserved. We tested this assumption by comparing intraspecies inter-tissue distances among pairs of species and found that the magnitude and relative ordering of these distances among tissues were conserved among mammals (Fig. 5). Future studies of the dynamics of inter-tissue matrices along different lineages might identify cases where particular tissues have become more or less specialized in their functions.

Analyses of more precise populations of cells than whole tissues will help to expose the biological bases underlying tissue diversity, but will be subject to many of the same pitfalls that we have highlighted. Some cell types will be more similar to each other than others and it is not yet clear whether technical variability between low-cell population studies will be sufficiently small for meta-analyses. Nevertheless, future evolutionary gene expression studies must seek to overcome these issues if we are to fully disentangle the developmental programs that govern vertebrate organismal diversity.

## Methods

### Read mapping

RNA-seq data from Brawand et al. [2], Merkin et al. [3], Lin et al. [6], and GTEx [13] were downloaded and mapped with STAR version 2.4.2a [19], to the following genome assemblies: musmus9, rhemac2, ratnor4, bostau4, galgal3, hg19, panTro2, ponAbe2, gorGor3, monDom5, and ornAna1. Gene annotations from Merkin et al. [3] were used for human, mouse, rat, macaque, chicken, and cow. Gene annotations from Ensembl release 61 were used for the remaining species. For the studies with biological replicates, the replicate with the longest reads was selected (if all replicates had the same read length, the most deeply sequenced replicate was used instead). A complete list of accessions can be found in Additional file 3: Table S1.

### Orthology definition

Gene orthologies were downloaded from Ensembl release 61. Amniote orthologs were defined as single-copy orthologous genes conserved in all 11 amniote species considered. Human-mouse orthologs were defined as single-copy genes conserved in human, mouse, and macaque.

### Gene expression analysis

The data were mapped with STAR using the –quantMode GeneCounts flag to obtain raw counts per gene. These values were then normalized by TMM normalization, using the edgeR package [15, 20]. Because TMM normalization rescales samples relative to one another, the data were re-normalized separately for each analysis.

### Clustering and distance metrics

Heat maps in Figs. 3f and 4c were clustered by average-linkage hierarchical clustering. The units bits^½^ are the result of taking the square root of the JSD, which scales this value so that it will satisfy the triangle inequality, converting the divergence to a distance metric [21].

## Additional files

Additional file 1: Figure S1. Multidimensional scaling analysis of samples from five datasets. Multidimensional scaling of samples assessed in this study plotted in two dimensions. Samples are colored by tissue, with the shape corresponding to the species. Within each shape is a one-letter code representing the dataset of origin. All samples were plotted for Brawand, Lin1, Lin2, and Merkin, along with corresponding tissues from GTex. (PDF 100 kb) Additional file 2: Figure S2. Clustering in PCA analysis is influenced by the composition of the dataset as a whole. **a** Heat map of the distances between samples from a simulated dataset. Simulated dataset includes seven tissues (T1–T7) from two species (Sp1, Sp2), where tissues T1–T4 are very similar to one another. **b** PCA analysis performed on the whole dataset—first two components shown. Tissues are designated by color, with four closely related tissues in different shades of blue. **c** PCA analysis performed on four diverse tissues, using T5–T7 and one representative tissue from T1–T4. **d** PCA analysis performed on four similar tissues, T1–T4. (PDF 149 kb) Additional file 3: Table S1. Contains a complete description of all samples and accessions used in this study including the Lin2 dataset, which is available through the ENCODE portal. (PDF 75 kb)
