# Supplementary figures and images for: Meta-analysis of RNA-seq expression data across species, tissues and studies
Source: Genome Biol. 2015 Dec 22;16:287. doi: 10.1186/s13059-015-0853-4 (PMC4699362; doi:10.1186/s13059-015-0853-4)

Figure S1

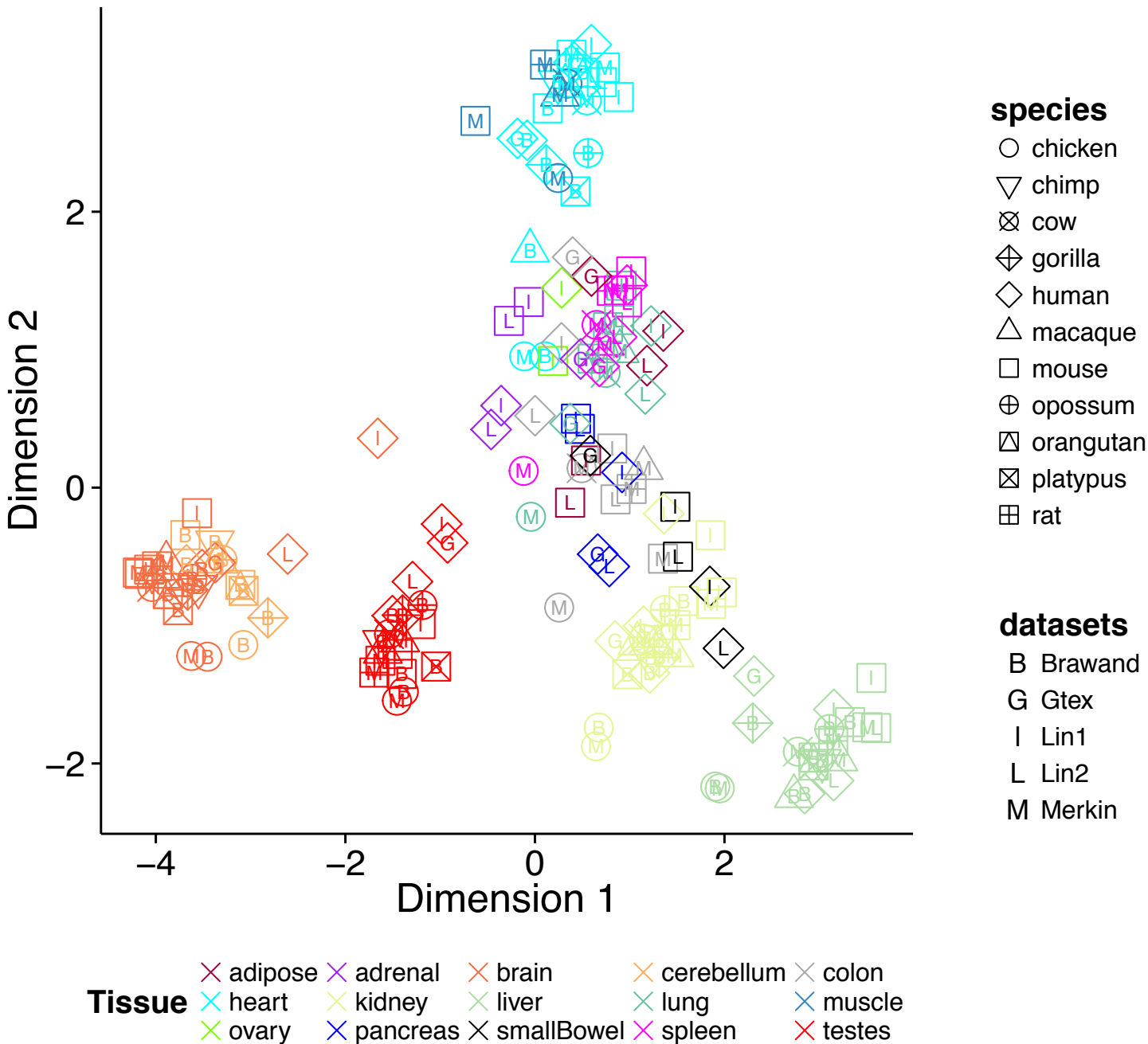

Supplement: Additional file 1: Figure S1. — Multidimensional scaling analysis of samples from five datasets. Multidimensional scaling of samples assessed in this study plotted in two dimensions. Samples are colored by tissue, with the shape corresponding to the species. Within each shape is a one-letter code representing the dataset of origin. All samples were plotted for Brawand, Lin1, Lin2, and Merkin, along with corresponding tissues from GTex. (PDF 100 kb) [file 13059_2015_853_MOESM1_ESM.pdf]

Figure S2

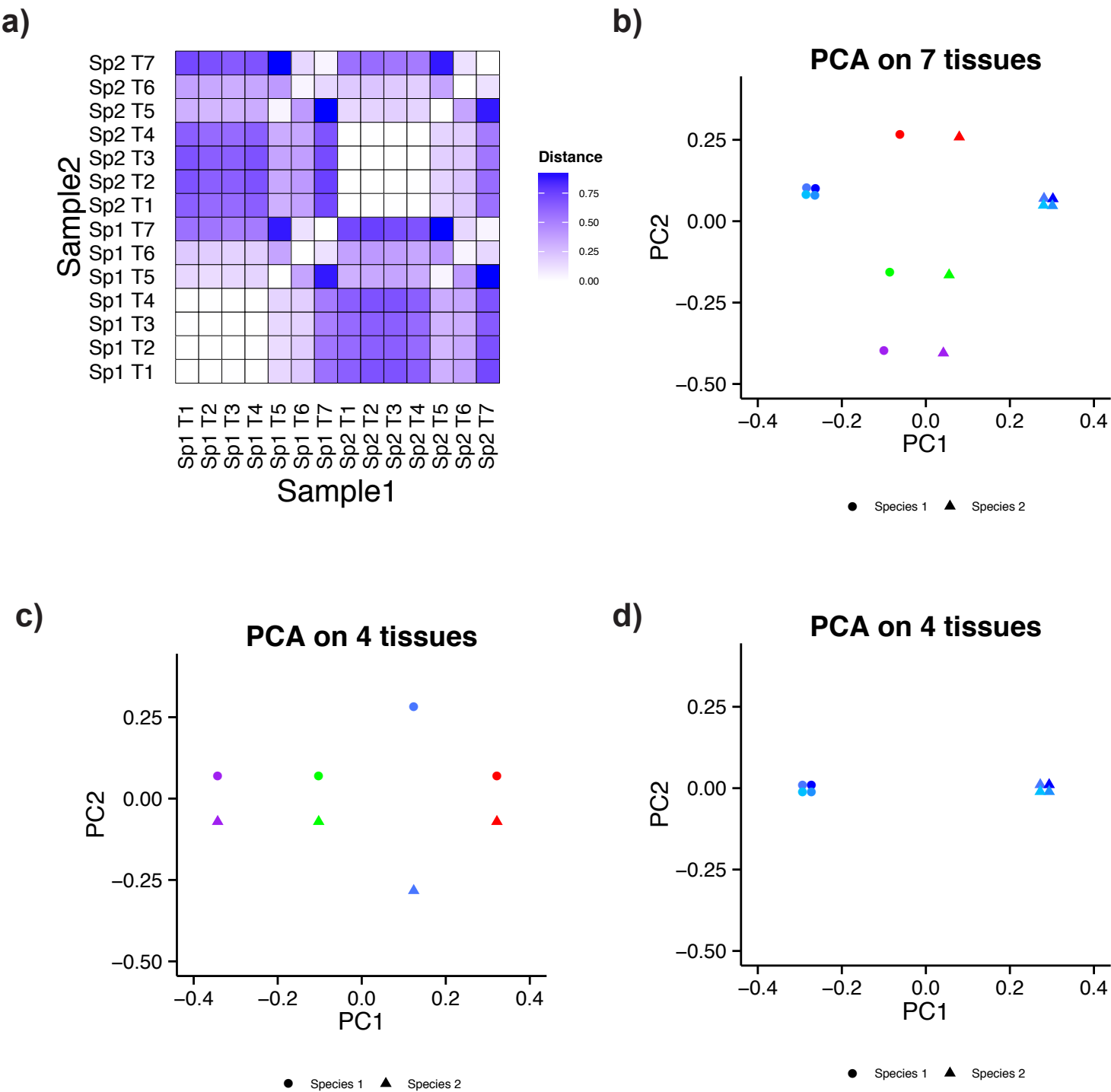

Supplement: Additional file 2: Figure S2. — Clustering in PCA analysis is influenced by the composition of the dataset as a whole. a Heat map of the distances between samples from a simulated dataset. Simulated dataset includes seven tissues (T1–T7) from two species (Sp1, Sp2), where tissues T1–T4 are very similar to one another. b PCA analysis performed on the whole dataset—first two components shown. Tissues are designated by color, with four closely related tissues in different shades of blue. c PCA analysis performed on four diverse tissues, using T5–T7 and one representative tissue from T1–T4. d PCA analysis performed on four similar tissues, T1–T4. (PDF 149 kb) [file 13059_2015_853_MOESM2_ESM.pdf]
