# Supplementary material for: Meta-analysis of RNA-seq expression data across species, tissues and studies
Source: Genome Biol. 2015 Dec 22;16:287. doi: 10.1186/s13059-015-0853-4 (PMC4699362; doi:10.1186/s13059-015-0853-4)
Supplement: Additional file 3: Table S1. — Contains a complete description of all samples and accessions used in this study including the Lin2 dataset, which is available through the ENCODE portal. (PDF 75 kb) [file 13059_2015_853_MOESM3_ESM.pdf]

Table S1. Description of all samples used in this meta-study - Lists study of origin, species, tissue (and sub-tissue if applicable), and accession numbers. Accession numbers with the prefix "ENC" can be accessed through the ENCODE portal at https://www.encodeproject.org/. All other accession numbers are available through http://www.ncbi.nlm.nih.gov/sra.

Study	Species	Organ	Experiment/project	Read accesssion(s)
Brawand	chicken	brain	SRX081870	SRR306711
Brawand	chicken	cerebellum	SRX081872	SRR306713
Brawand	chicken	heart	SRX081874	SRR306715
Brawand	chicken	kidney	SRX081876	SRR306717
Brawand	chicken	liver	SRX081878	SRR306719,SRR306720
Brawand	chicken	testis	SRX081880	SRR306723
Brawand	chimpanzee	brain	SRX081960	SRR306816
Brawand	chimpanzee	cerebellum	SRX081962	SRR306818
Brawand	chimpanzee	heart	SRX081964	SRR306820
Brawand	chimpanzee	kidney	SRX081966	SRR306822
Brawand	chimpanzee	liver	SRX081968	SRR306824
Brawand	chimpanzee	testis	SRX081969	SRR306825
Brawand	gorilla	brain	SRX081945	SRR306801
Brawand	gorilla	cerebellum	SRX081947	SRR306803
Brawand	gorilla	heart	SRX081949	SRR306805
Brawand	gorilla	kidney	SRX081951	SRR306807
Brawand	gorilla	liver	SRX081953	SRR306809
Brawand	gorilla	testis	SRX081954	SRR306810
Brawand	human	brain	SRX081984	SRR306840
Brawand	human	cerebellum	SRX081989	SRR306845,SRR306846
Brawand	human	heart	SRX081991	SRR306848,SRR306849
Brawand	human	kidney	SRX081995	SRR306853
Brawand	human	liver	SRX081996	SRR306854,SRR306855
Brawand	human	testis	SRX081999	SRR306858
Brawand	macaque	brain	SRX081924	SRR306779
Brawand	macaque	cerebellum	SRX081926	SRR306781
Brawand	macaque	heart	SRX081928	SRR306783
Brawand	macaque	kidney	SRX081930	SRR306785
Brawand	macaque	liver	SRX081932	SRR306787,SRR306788
Brawand	macaque	testis	SRX081934	SRR306790

Brawand	mouse	brain	SRX081906	SRR306758,SRR306759,SRR306760,SRR306761
Brawand	mouse	cerebellum	SRX081910	SRR306765
Brawand	mouse	heart	SRX081913	SRR306768
Brawand	mouse	kidney	SRX081916	SRR306771
Brawand	mouse	liver	SRX081919	SRR306774
Brawand	mouse	testis	SRX081921	SRR306776
Brawand	opossum	brain	SRX081894	SRR306744
Brawand	opossum	cerebellum	SRX081896	SRR306746
Brawand	opossum	heart	SRX081898	SRR306749,SRR306750
Brawand	opossum	kidney	SRX081900	SRR306752
Brawand	opossum	liver	SRX081902	SRR306754
Brawand	opossum	testis	SRX081904	SRR306756
Brawand	orangutan	brain	SRX081936	SRR306792
Brawand	orangutan	cerebellum	SRX081937	SRR306793
Brawand	orangutan	heart	SRX081939	SRR306795
Brawand	orangutan	kidney	SRX081941	SRR306797
Brawand	orangutan	liver	SRX081943	SRR306799
Brawand	platypus	brain	SRX081882	SRR306726,SRR306727
Brawand	platypus	cerebellum	SRX081884	SRR306729
Brawand	platypus	heart	SRX081886	SRR306731
Brawand	platypus	kidney	SRX081888	SRR306733,SRR306734
Brawand	platypus	liver	SRX081890	SRR306737,SRR306738
Brawand	platypus	testis	SRX081892	SRR306740,SRR306741
Merkin	chicken	brain	SRX196371	SRR594500
Merkin	chicken	colon	SRX196372	SRR594501
Merkin	chicken	heart	SRX196373	SRR594502
Merkin	chicken	kidney	SRX196374	SRR594503
Merkin	chicken	liver	SRX196375	SRR594504
Merkin	chicken	lung	SRX196376	SRR594505
Merkin	chicken	skeletal muscle	SRX196377	SRR594506
Merkin	chicken	spleen	SRX196378	SRR594507
Merkin	chicken	testis	SRX196379	SRR594508
Merkin	cow	brain	SRX196344	SRR594473
Merkin	cow	colon	SRX196345	SRR594474
Merkin	cow	heart	SRX196346	SRR594475

Merkin	cow	kidney	SRX196347	SRR594476
Merkin	cow	liver	SRX196348	SRR594477
Merkin	cow	lung	SRX196349	SRR594478
Merkin	cow	skeletal muscle	SRX196350	SRR594479
Merkin	cow	spleen	SRX196351	SRR594480
Merkin	cow	testis	SRX196352	SRR594481
Merkin	macaque	brain	SRX196326	SRR594455
Merkin	macaque	colon	SRX196318	SRR594447
Merkin	macaque	heart	SRX196319	SRR594448
Merkin	macaque	kidney	SRX196329	SRR594458
Merkin	macaque	liver	SRX196330	SRR594459
Merkin	macaque	lung	SRX196331	SRR594460
Merkin	macaque	skeletal muscle	SRX196323	SRR594452
Merkin	macaque	spleen	SRX196333	SRR594462
Merkin	macaque	testis	SRX196334	SRR594463
Merkin	mouse	brain	SRX196273	SRR594402
Merkin	mouse	colon	SRX196274	SRR594403
Merkin	mouse	heart	NA	NA
Merkin	mouse	kidney	SRX196275	SRR594404
Merkin	mouse	liver	SRX196276	SRR594405
Merkin	mouse	lung	SRX196277	SRR594406
Merkin	mouse	skeletal muscle	SRX196278	SRR594407
Merkin	mouse	spleen	SRX196279	SRR594408
Merkin	mouse	testis	SRX196280	SRR594409
Merkin	rat	brain	SRX196299	SRR594428
Merkin	rat	colon	SRX196300	SRR594429
Merkin	rat	heart	SRX196301	SRR594430
Merkin	rat	kidney	SRX196302	SRR594431
Merkin	rat	liver	SRX196303	SRR594432
Merkin	rat	lung	SRX196304	SRR594433
Merkin	rat	skeletal muscle	SRX196305	SRR594434
Merkin	rat	spleen	SRX196306	SRR594435
Merkin	rat	testis	SRX196307	SRR594436
Lin1	human	adipose	ENCSR236OON	ENCFF170RHF,ENCFF437XFH
Lin1	human	adrenal	ENCSR680AAZ	ENCFF028DUO,ENCFF470RWW

Lin1	human	brain	ENCSR274JRR	ENCFF850ZLY,ENCFF897IUQ
Lin1	human	heart	ENCSR635GTY	ENCFF770NYA,ENCFF076IRZ
Lin1	human	kidney	ENCSR071ZMO	ENCFF912LIX,ENCFF057RDO
Lin1	human	liver	ENCSR085HNI	ENCFF187OKV,ENCFF283RUU
Lin1	human	lung	ENCSR129KCJ	ENCFF433PKC,ENCFF546XEW
Lin1	human	ovary	ENCSR046XHI	ENCFF419GVS,ENCFF135CVY
Lin1	human	pancreas	ENCSR001UXR	ENCFF576OBS,ENCFF063OPQ
Lin1	human	sigmoid colon	ENCSR270OKS	ENCFF734ZAD,ENCFF261RWK
Lin1	human	small bowel	ENCSR612HYR	ENCFF338DKW,ENCFF721RGF
Lin1	human	spleen	ENCSR448VSW	ENCFF482WYS,ENCFF058MGQ
Lin1	human	testis	ENCSR693GGB	ENCFF016TGP,ENCFF604DIX
Lin1	mouse	adipose	ENCSR288TLO	ENCFF547BPL,ENCFF554BIM
Lin1	mouse	adrenal	ENCSR713OCQ	ENCFF563FDS,ENCFF470ULL
Lin1	mouse	brain	ENCSR554PHF	ENCFF286WTQ,ENCFF358NPU
Lin1	mouse	heart	ENCSR164BAZ	ENCFF871XHK,ENCFF952JKH
Lin1	mouse	kidney	ENCSR394YLM	ENCFF445EZT,ENCFF345AQQ
Lin1	mouse	liver	ENCSR216KLZ	ENCFF161LEK,ENCFF516HOO
Lin1	mouse	lung	ENCSR870AQU	ENCFF276XMW,ENCFF361ASL
Lin1	mouse	ovary	ENCSR516UNF	ENCFF463WEH,ENCFF312OKA
Lin1	mouse	pancreas	ENCSR248XKS	ENCFF955YPL,ENCFF589FKL
Lin1	mouse	sigmoid	ENCSR518GDK	ENCFF279LVO,ENCFF400OMQ
Lin1	mouse	small bowel	ENCSR170SVO	ENCFF996KVO,ENCFF269CEX
Lin1	mouse	spleen	ENCSR966JPL	ENCFF848QCK,ENCFF511PCY
Lin1	mouse	testis	ENCSR266ESZ	ENCFF786ZKB,ENCFF517RDO
Lin2	human	adipose	ENCSR236OON	ENCFF592VVB,ENCFF359HIQ
Lin2	human	adrenal	ENCSR680AAZ	ENCFF709FHN,ENCFF681HNP
Lin2	human	brain	ENCSR274JRR	ENCFF456MMS,ENCFF716WNR
Lin2	human	heart	ENCSR635GTY	ENCFF464TEM,ENCFF221QNJ
Lin2	human	kidney	ENCSR071ZMO	ENCFF044VER,ENCFF640PYL
Lin2	human	liver	ENCSR085HNI	ENCFF650JAM,ENCFF803DXA
Lin2	human	lung	ENCSR129KCJ	ENCFF911WYB,ENCFF723QXK
Lin2	human	pancreas	ENCSR001UXR	ENCFF541KUW,ENCFF386MOY
Lin2	human	sigmoid colon	ENCSR270OKS	ENCFF322RPT,ENCFF782AHJ
Lin2	human	small bowel	ENCSR612HYR	ENCFF540SNP,ENCFF999PRA
Lin2	human	spleen	ENCSR448VSW	ENCFF926YPC,ENCFF111IRS

Lin2	human	testis	ENCSR693GGB	ENCFF034BHU,ENCFF788AET
Lin2	mouse	adipose	ENCSR288TLO	ENCFF128KGA,ENCFF510DLJ
Lin2	mouse	adrenal	ENCSR713OCQ	ENCFF307YNT,ENCFF731TMT
Lin2	mouse	brain	ENCSR554PHF	ENCFF445AWP,ENCFF958CHE
Lin2	mouse	heart	ENCSR164BAZ	ENCFF104UFH,ENCFF126WYO
Lin2	mouse	kidney	ENCSR394YLM	ENCFF214JLE,ENCFF883OGF
Lin2	mouse	liver	ENCSR216KLZ	ENCFF492PRP,ENCFF581OEV
Lin2	mouse	lung	ENCSR870AQU	ENCFF146GNS,ENCFF280IUI
Lin2	mouse	pancreas	ENCSR248XKS	ENCFF849WWH,ENCFF224IMT
Lin2	mouse	sigmoid	ENCSR518GDK	ENCFF631QWX,ENCFF016KHB
Lin2	mouse	small bowel	ENCSR170SVO	ENCFF933LCA,ENCFF684WUW
Lin2	mouse	spleen	ENCSR966JPL	ENCFF432KKN,ENCFF859JTH
Lin2	mouse	testis	ENCSR266ESZ	ENCFF682XSC,ENCFF690HKC
Gtex	human	Adipose-Subcutaneous	SRP012682	SRR1081567
Gtex	human	Adipose-Subcutaneous	SRP012683	SRR1083311
Gtex	human	Adipose-Subcutaneous	SRP012684	SRR665551
Gtex	human	Adipose-VisceralOmentum	SRP012685	SRR1070713
Gtex	human	Adipose-VisceralOmentum	SRP012686	SRR1094457
Gtex	human	Adipose-VisceralOmentum	SRP012687	SRR1083918
Gtex	human	AdrenalGland	SRP012688	SRR1088461
Gtex	human	AdrenalGland	SRP012689	SRR1088365
Gtex	human	AdrenalGland	SRP012690	SRR1120913
Gtex	human	Artery-Aorta	SRP012691	SRR1101591
Gtex	human	Artery-Aorta	SRP012692	SRR1086393
Gtex	human	Artery-Aorta	SRP012693	SRR808351
Gtex	human	Artery-Coronary	SRP012694	SRR813471
Gtex	human	Artery-Coronary	SRP012695	SRR1089375
Gtex	human	Artery-Coronary	SRP012696	SRR1096736
Gtex	human	Artery-Tibial	SRP012697	SRR817094
Gtex	human	Artery-Tibial	SRP012698	SRR1089974
Gtex	human	Artery-Tibial	SRP012699	SRR613568
Gtex	human	Bladder	SRP012700	SRR1086236
Gtex	human	Bladder	SRP012701	SRR1079830
Gtex	human	Bladder	SRP012702	SRR1084917
Gtex	human	Brain-Amygdala	SRP012703	SRR1085015

Gtex	human	Brain-Amygdala	SRP012704	SRR1095865
Gtex	human	Brain-Amygdala	SRP012705	SRR661818
Gtex	human	Brain-AnteriorCingulateCortexBA24	SRP012706	SRR1073143
Gtex	human	Brain-AnteriorCingulateCortexBA24	SRP012707	SRR661133
Gtex	human	Brain-AnteriorCingulateCortexBA24	SRP012708	SRR814989
Gtex	human	Brain-CaudateBasalGanglia	SRP012709	SRR602271
Gtex	human	Brain-CaudateBasalGanglia	SRP012710	SRR657731
Gtex	human	Brain-CaudateBasalGanglia	SRP012711	SRR663261
Gtex	human	Brain-CerebellarHemisphere	SRP012712	SRR608718
Gtex	human	Brain-CerebellarHemisphere	SRP012713	SRR1080927
Gtex	human	Brain-CerebellarHemisphere	SRP012714	SRR1098519
Gtex	human	Brain-Cerebellum	SRP012715	SRR627299
Gtex	human	Brain-Cerebellum	SRP012716	SRR659412
Gtex	human	Brain-Cerebellum	SRP012717	SRR662871
Gtex	human	Brain-Cortex	SRP012718	SRR664854
Gtex	human	Brain-Cortex	SRP012719	SRR627449
Gtex	human	Brain-Cortex	SRP012720	SRR816770
Gtex	human	Brain-FrontalCortexBA9	SRP012721	SRR658307
Gtex	human	Brain-FrontalCortexBA9	SRP012722	SRR657777
Gtex	human	Brain-FrontalCortexBA9	SRP012723	SRR661349
Gtex	human	Brain-Hippocampus	SRP012724	SRR656564
Gtex	human	Brain-Hippocampus	SRP012725	SRR661255
Gtex	human	Brain-Hippocampus	SRP012726	SRR614814
Gtex	human	Brain-Hypothalamus	SRP012727	SRR608230
Gtex	human	Brain-Hypothalamus	SRP012728	SRR660091
Gtex	human	Brain-Hypothalamus	SRP012729	SRR661179
Gtex	human	Brain-NucleusAccumbensBasalGanglia	SRP012730	SRR602808
Gtex	human	Brain-NucleusAccumbensBasalGanglia	SRP012731	SRR1087031
Gtex	human	Brain-NucleusAccumbensBasalGanglia	SRP012732	SRR655134
Gtex	human	Brain-PutamenBasalGanglia	SRP012733	SRR598894
Gtex	human	Brain-PutamenBasalGanglia	SRP012734	SRR614515
Gtex	human	Brain-PutamenBasalGanglia	SRP012735	SRR665563
Gtex	human	Brain-SpinalCordCervicalC-1	SRP012736	SRR602598
Gtex	human	Brain-SpinalCordCervicalC-1	SRP012737	SRR612407
Gtex	human	Brain-SpinalCordCervicalC-1	SRP012738	SRR613807

Gtex	human	Brain-SubstantiaNigra	SRP012739	SRR599486
Gtex	human	Brain-SubstantiaNigra	SRP012740	SRR662138
Gtex	human	Brain-SubstantiaNigra	SRP012741	SRR817797
Gtex	human	Breast-MammaryTissue	SRP012742	SRR1079948
Gtex	human	Breast-MammaryTissue	SRP012743	SRR1070738
Gtex	human	Breast-MammaryTissue	SRP012744	SRR1084674
Gtex	human	Cells-EBV-transformedLymphocytes	SRP012745	SRR818009
Gtex	human	Cells-EBV-transformedLymphocytes	SRP012746	SRR811309
Gtex	human	Cells-EBV-transformedLymphocytes	SRP012747	SRR819234
Gtex	human	Cells-LeukemiaCellLineCML	SRP012748	SRR1077334
Gtex	human	Cells-LeukemiaCellLineCML	SRP012749	SRR612587
Gtex	human	Cells-LeukemiaCellLineCML	SRP012750	SRR613294
Gtex	human	Cells-TransformedFibroblasts	SRP012751	SRR1069121
Gtex	human	Cells-TransformedFibroblasts	SRP012752	SRR1086094
Gtex	human	Cells-TransformedFibroblasts	SRP012753	SRR1091206
Gtex	human	Cervix-Ectocervix	SRP012754	SRR1088832
Gtex	human	Cervix-Ectocervix	SRP012755	SRR1097035
Gtex	human	Cervix-Ectocervix	SRP012756	SRR1097574
Gtex	human	Cervix-Endocervix	SRP012757	SRR1077239
Gtex	human	Cervix-Endocervix	SRP012758	SRR1096057
Gtex	human	Cervix-Endocervix	SRP012759	SRR1098612
Gtex	human	Colon-Sigmoid	SRP012760	SRR1093366
Gtex	human	Colon-Sigmoid	SRP012761	SRR1091524
Gtex	human	Colon-Sigmoid	SRP012762	SRR1102998
Gtex	human	Colon-Transverse	SRP012763	SRR1098098
Gtex	human	Colon-Transverse	SRP012764	SRR1099451
Gtex	human	Colon-Transverse	SRP012765	SRR816794
Gtex	human	Esophagus-GastroesophagealJunction	SRP012766	SRR1101717
Gtex	human	Esophagus-GastroesophagealJunction	SRP012767	SRR1087127
Gtex	human	Esophagus-GastroesophagealJunction	SRP012768	SRR1100005
Gtex	human	Esophagus-Mucosa	SRP012769	SRR1077310
Gtex	human	Esophagus-Mucosa	SRP012770	SRR1085211
Gtex	human	Esophagus-Mucosa	SRP012771	SRR1072055
Gtex	human	Esophagus-Muscularis	SRP012772	SRR1078514
Gtex	human	Esophagus-Muscularis	SRP012773	SRR1089078

Gtex	human	Esophagus-Muscularis	SRP012774	SRR815446
Gtex	human	FallopianTube	SRP012775	SRR1071359
Gtex	human	FallopianTube	SRP012776	SRR1082520
Gtex	human	FallopianTube	SRP012777	SRR1083776
Gtex	human	Heart-AtrialAppendage	SRP012778	SRR1093243
Gtex	human	Heart-AtrialAppendage	SRP012779	SRR818530
Gtex	human	Heart-AtrialAppendage	SRP012780	SRR808327
Gtex	human	Heart-LeftVentricle	SRP012781	SRR598509
Gtex	human	Heart-LeftVentricle	SRP012782	SRR815517
Gtex	human	Heart-LeftVentricle	SRP012783	SRR602461
Gtex	human	Kidney-Cortex	SRP012784	SRR1071807
Gtex	human	Kidney-Cortex	SRP012785	SRR1105272
Gtex	human	Kidney-Cortex	SRP012786	SRR809943
Gtex	human	Liver	SRP012787	SRR1090556
Gtex	human	Liver	SRP012788	SRR1101883
Gtex	human	Liver	SRP012789	SRR807995
Gtex	human	Lung	SRP012790	SRR1081283
Gtex	human	Lung	SRP012791	SRR662162
Gtex	human	Lung	SRP012792	SRR817166
Gtex	human	MinorSalivaryGland	SRP012793	SRR1081589
Gtex	human	MinorSalivaryGland	SRP012794	SRR1078392
Gtex	human	MinorSalivaryGland	SRP012795	SRR1097245
Gtex	human	Muscle-Skeletal	SRP012796	SRR615273
Gtex	human	Muscle-Skeletal	SRP012797	SRR612803
Gtex	human	Muscle-Skeletal	SRP012798	SRR820907
Gtex	human	Nerve-Tibial	SRP012799	SRR612911
Gtex	human	Nerve-Tibial	SRP012800	SRR807613
Gtex	human	Nerve-Tibial	SRP012801	SRR821124
Gtex	human	Ovary	SRP012802	SRR1071475
Gtex	human	Ovary	SRP012803	SRR1102005
Gtex	human	Ovary	SRP012804	SRR814293
Gtex	human	Pancreas	SRP012805	SRR1091032
Gtex	human	Pancreas	SRP012806	SRR1073167
Gtex	human	Pancreas	SRP012807	SRR1081259
Gtex	human	Pituitary	SRP012808	SRR1077708

Gtex	human	Pituitary	SRP012809	SRR1077968
Gtex	human	Pituitary	SRP012810	SRR821573
Gtex	human	Prostate	SRP012811	SRR1086869
Gtex	human	Prostate	SRP012812	SRR1092444
Gtex	human	Prostate	SRP012813	SRR1099402
Gtex	human	Skin-NotSunExposedSuprapubic	SRP012814	SRR1069048
Gtex	human	Skin-NotSunExposedSuprapubic	SRP012815	SRR1087801
Gtex	human	Skin-NotSunExposedSuprapubic	SRP012816	SRR659756
Gtex	human	Skin-SunExposedLowerLeg	SRP012817	SRR615587
Gtex	human	Skin-SunExposedLowerLeg	SRP012818	SRR655471
Gtex	human	Skin-SunExposedLowerLeg	SRP012819	SRR807775
Gtex	human	SmallIntestine-TerminalIleum	SRP012820	SRR1072602
Gtex	human	SmallIntestine-TerminalIleum	SRP012821	SRR1093314
Gtex	human	SmallIntestine-TerminalIleum	SRP012822	SRR1094531
Gtex	human	Spleen	SRP012823	SRR1085087
Gtex	human	Spleen	SRP012824	SRR1069303
Gtex	human	Spleen	SRP012825	SRR1086915
Gtex	human	Stomach	SRP012826	SRR1088878
Gtex	human	Stomach	SRP012827	SRR814268
Gtex	human	Stomach	SRP012828	SRR1076268
Gtex	human	Testis	SRP012829	SRR1068788
Gtex	human	Testis	SRP012830	SRR1080022
Gtex	human	Testis	SRP012831	SRR1081449
Gtex	human	Thyroid	SRP012832	SRR1092567
Gtex	human	Thyroid	SRP012833	SRR661409
Gtex	human	Thyroid	SRP012834	SRR808886
Gtex	human	Uterus	SRP012835	SRR1077211
Gtex	human	Uterus	SRP012836	SRR1082128
Gtex	human	Uterus	SRP012837	SRR820026
Gtex	human	Vagina	SRP012838	SRR1093410
Gtex	human	Vagina	SRP012839	SRR1095599
Gtex	human	Vagina	SRP012840	SRR1101907
Gtex	human	WholeBlood	SRP012841	SRR627423
Gtex	human	WholeBlood	SRP012842	SRR808728
Gtex	human	WholeBlood	SRP012843	SRR813339
